# The Sphygmograph for Diagnosis, Peculiarly Slow Pulse

**Published:** 1879-08

**Authors:** 


### THE SPHYGMOGRAPH FOR DIAGNOSIS, PECULIARLY SLOW PULSE.

(*Proceedings Baltimore Academy of Medicine.*)

Dr. C. Johnston, exhibited some beautiful sphygmographic tracings, showing the value of this new instrument in diagnosis; one case was of special interest. A lady patient had a pulse of

33 to the minute, which had attracted his attention from its peculiar slowness. From time to time he had tested the pulse with the utmost care, but could never get more than the 33 beats. The sphygmographic tracing exhibited a very slight elevation in the middle of each trace division, in evidence that a feeble heart-pulse did occur with every alternate strong beat, too feeble for the fingers to detect, and yet clearly defined by the sphygmographic tracings.

Under a tonic treatment, the lady improved much, and, in course of a month, the pulse of 33 to the minute had developed into 66 equal pulsations. The sphygmograph now gave the 66 equally strong wave impulses to the minute. The pulse had always been 66—the alternate beats, however, having been too feeble to be recognized, thus giving the explanation of the very slow pulse.—*The Virginia Medical Monthly, July, 1879.*

